# The role of empathy in choosing rewards from another's perspective

**DOI:** 10.3389/fnhum.2013.00174

**Published:** 2013-05-23

**Authors:** Garret O'Connell, Anastasia Christakou, Anthony T. Haffey, Bhismadev Chakrabarti

**Affiliations:** School of Psychology and Clinical Language Sciences, Centre for Integrative Neuroscience and Neurodynamics, University of ReadingReading, UK

**Keywords:** empathy, reward, temporal discounting, social distance, simulation

## Abstract

As social animals, we regularly act in the interest of others by making decisions on their behalf. These decisions can take the form of choices between smaller short-term rewards and larger long-term rewards, and can be effectively indexed by temporal discounting (TD). In a TD paradigm, a reward loses subjective value with increasing delay presumably because it becomes more difficult to simulate how much the recipient (e.g., future self) will value it. If this is the case, then the value of delayed rewards should be discounted even more steeply when we are choosing for someone whose feelings we do not readily simulate, such as socially distant strangers. Second, the ability to simulate shows individual differences and is indexed by trait empathy. We hypothesized that individuals high in trait empathy will more readily simulate, and hence discount less steeply for distant others, compared to those who are low on trait empathy. To test these predictions, we asked 63 participants from the general population to perform a TD task from the perspectives of close and distant others, as well as their own. People were found to discount less steeply for themselves, and the steepness of TD increased with increasing distance from self. Additionally, individuals who scored high in trait empathy were found to discount less steeply for distant others compared to those who scored low. These findings confirm the role of empathy in determining how we choose rewards for others.

## Introduction

As social beings, we do not just make decisions for ourselves, but regularly have to make decisions on behalf of others. We invest great effort into judging what someone else would like when buying gifts, or making plans for them. Consider the case of a husband trying to decide what his wife would prefer: a fancy dinner out this evening or a weekend trip away in 2 weeks' time. How do we make such decisions? A body of literature on how we make choices for ourselves shows that a key role in these decisions is played by our emotional state (Damasio et al., 1991). If our own emotions are crucial to making choices for ourselves, it follows that we need a good understanding of another person's emotions and mental states in order to make choices on their behalf. Empathy is a trait that quantifies this capacity to understand others' emotions and mental states and respond appropriately to them (for a review, see Chakrabarti et al., 2006). In the current paper, we examine the role of empathy in making choices on another's behalf in an intertemporal context.

One of the most commonly encountered choices are those between short-term and long-term rewards. Such intertemporal preferences are indexed by temporal discounting (TD). In a typical TD paradigm, a series of choices between smaller immediate and larger delayed monetary amounts are presented. The commonly observed response pattern is that with increasing delay, the more immediate though lesser rewards are preferred over larger, later rewards. The rate at which rewards are subjectively devalued slows down as delay increases, resulting in a steep-to-flat “discounting curve,” suggesting that rewards are devalued with time more rapidly over shorter delays than longer delays (Ainslie, 1975). This discounting function has been associated with intelligence (Mischel and Metzner, 1962; Kirby et al., 2005; Shamosh et al., 2008), impulsivity (Bickel et al., 1999; de Wit et al., 2007; Christakou et al., 2011), and consequential life outcomes such as health, wealth and social-functioning (Mischel et al., 1989; Moffitt et al., 2011). While predictors of how individuals discount when they have to make choices about themselves have been well investigated, little research has focused on the discounting functions for others.

It has been suggested that we devalue delayed rewards because we empathise less with the feelings of their recipient (i.e., future selves) (Loewenstein, 1996). A key process underlying empathy is that of simulation, i.e., the ability to put ourselves in the shoes of others (Gordon, 1992; Shanton and Goldman, 2010). Simulation provides a potential mechanism to understand how another person feels by imagining how we ourselves would feel in their situation, and has been proposed to underlie theory of mind (Shanton and Goldman, 2010). This mechanism applies equally to ourselves, i.e., we put ourselves in the shoes of our future selves, to predict how we will feel in the future. Recent functional neuroimaging studies provide indirect evidence for simulation, by showing involvement of the ventromedial prefrontal cortex in making value-based decisions for self as well as for others (Nicolle et al., 2012; Suzuki et al., 2012; Janowski et al., 2013). Neural and other indices of simulation (e.g., vicarious pain responses) are greater if the person is a socially close one (i.e., familiar or liked) than if the person is socially distant (Singer et al., 2006; Xu et al., 2009; Cheng et al., 2010). Arguably, simulation is easiest if the person to simulate is one's own self (minimum social distance). Social distance could thus be viewed as a proxy measure for ease of simulation.

**Figure 1:**
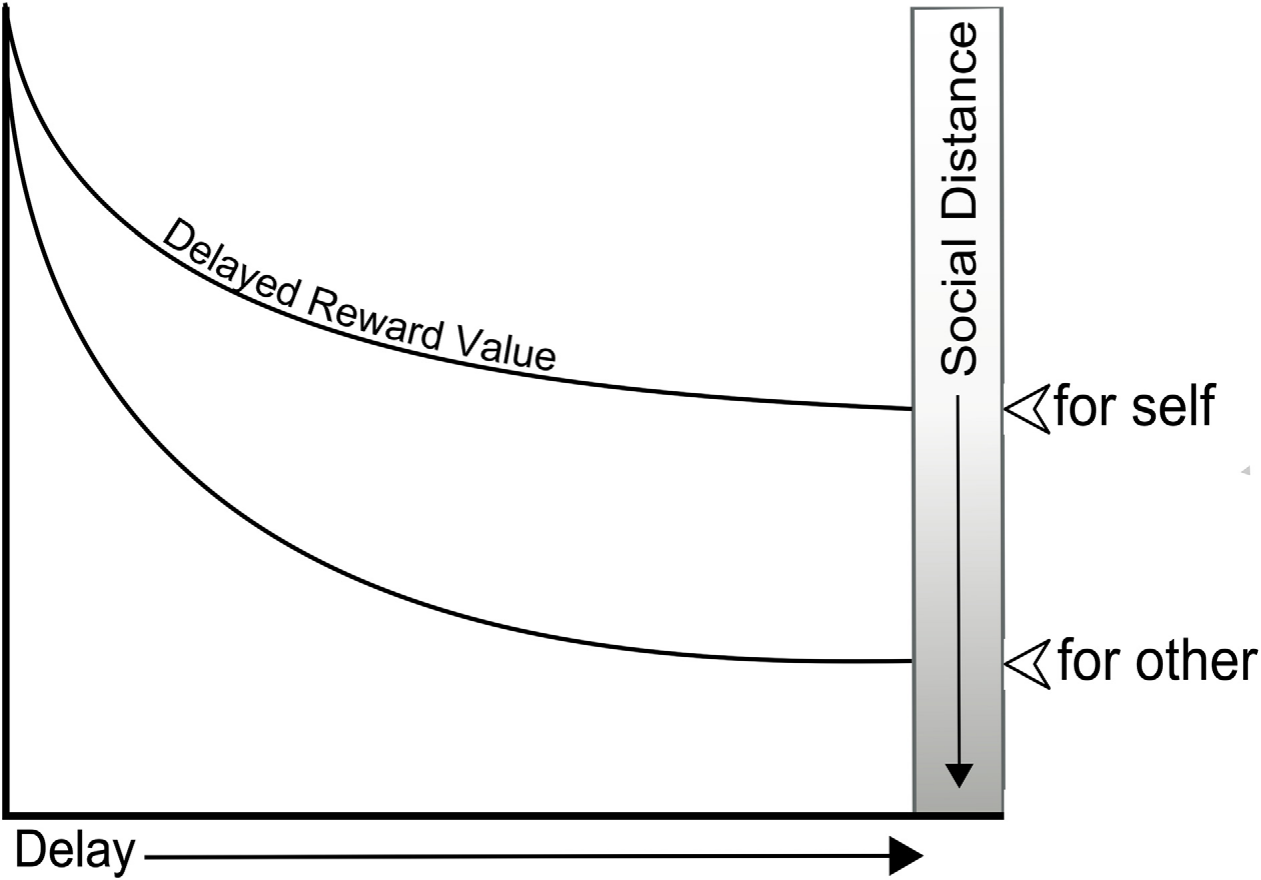
**Diagram of the hypothesized effect of the social distance of others on the temporal discounting of rewards for them**.

The above effect has direct implications for TD for self and others. For the self, it suggests that increasing the delay to reward reduces empathy for the recipient by increasing their social distance. This was supported by a set of studies by Bartels and Rips (2010) showing that social distance (operationalized by the authors as “psychological connectedness”) with the future self was directly proportional to the rate of discounting. For the other, it predicts that people will tend to discount less steeply for themselves and close others (who are easy to simulate), compared to distant others (who are difficult to simulate) (see Figure 1).

The ability to put one's self in another's place and simulate their feelings is indexed by trait empathy. If TD changes as a function of simulation, it is expected that highly empathic people (who simulate easily) will discount less steeply when making choices on behalf of others. As a corollary, people low in empathy will find it difficult to simulate distant others, and hence will discount more steeply when making choices on their behalf. To test these predictions, we examined TD from the perspective of others at different social distances. It is important to note that this is not equivalent to *social discounting*, in which rewards for others are discounted between close and distant others with no delay (Jones and Rachlin, 2006).

Specifically, we predicted that:

1. TD for others will be steeper than for self and will increase with increasing social distance (i.e., the relative steepness of discounting will vary as follows: distant other > close other > self).
2. Trait empathy will be associated negatively with the steepness of discounting for distant others (i.e., highly empathic people will discount less steeply for distant others).

## Materials and methods

### Participants

76 participants (38 female; age: *M* = 24.7 years, *SD* = 1.52), drawn largely from the university student population, consented to participate and received £6 for their time. An exclusion criterion was being a non-native English speaker. This study was approved by the University of Reading Research Ethics Committee.

### Trait empathy measures

Participants completed online versions of the Empathy Quotient (EQ; Baron-Cohen and Wheelwright, 2004) and the Interpersonal Reactivity Index (IRI; Davis, 1980). The personal distress subscale of the IRI was omitted as it was not directly relevant to this study.

### Social distance procedure

A social distance procedure was used to identify close and distant others. This task measures perceptions of others across dimensions of familiarity, similarity and kinship (Liviatan et al., 2008; Osiński, 2009). Participants were first instructed to list persons they know in descending order of familiarity between 1 and 100 at selected positions [as described in (Jones and Rachlin, 2006)]. Persons identified at the 4th and 43rd positions were used as close and distant others respectively, based on the observation that these points covered the maximum rate of change of the social discounting curve in a previous report (Jones and Rachlin, 2006). There were no restrictions on the category of relationship that could be used for these positions (e.g., spouse, sibling, friend), nor was this data collected.

### Temporal discounting task

For this task, the following instructions were given by the experimenter:

“This task involves a series of choices between smaller amounts of money now or larger amounts of money later. However, you will also be asked to perform the task as if you were someone you know. Try and put yourself in their shoes and imagine how they would respond.”

Participants were told to try and not to factor in particulars about their or the others' financial situation, only to select the preferred option. Because of the current study's focus on empathy, participants were instructed to make the decision *from the perspective* of the other, rather than *for the benefit* of other, which is more akin to altruism. To avoid self-bias (i.e., participants resorting to responding with their own preferences without considering how others might differ), decisions for self-took place after decisions for others had been completed, as suggested by Faro and Rottenstreich (2006).

**Figure 2:**
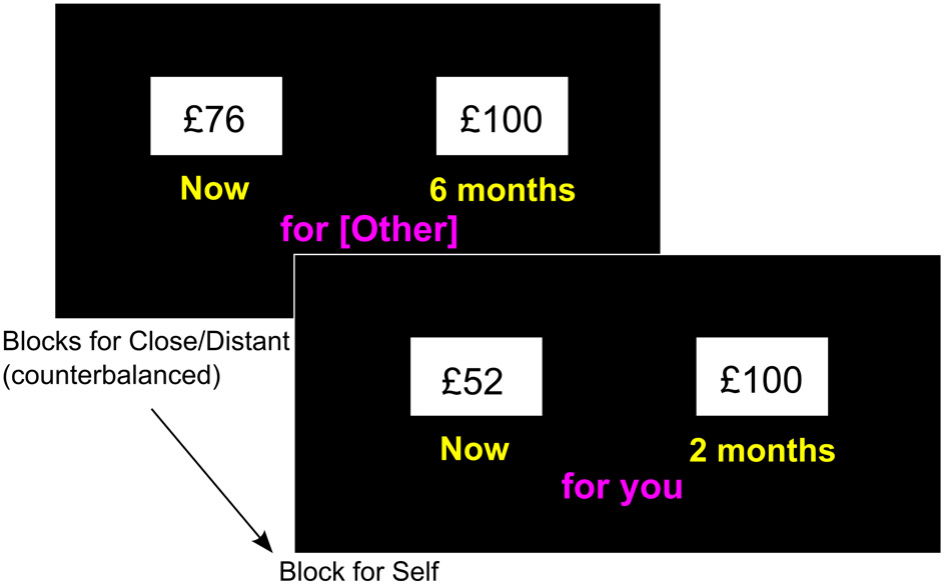
**Task design and an example trial from each social condition block (note: delay, immediate amount and sides on which amounts are presented vary within blocks)**.

The order of blocks (one each) for close and distant conditions was counterbalanced across participants. The task was run using E-Prime version 2.0. The person's perspective from whom the task was to be performed was shown before every trial and under the options during selection. Task options were between a variable immediate amount (<£100) or £100 at one of a randomly ordered sequence of 6 delays (weeks: 1, 3; months: 2, 5, 9, 18). Both immediate and delayed amounts were presented together and the participant selected the left or right amount with a keystroke (see Figure 2). The sides on which the immediate and delayed rewards were presented were counterbalanced across participants. The double-limits algorithm was used to estimate the variable amount (Johnson and Bickel, 2002) and indifference points (i.e., immediate amount value at which choices of £100 at a given delay were equally likely) used to map TD curves. Rate of TD was estimated using the area-under-the-curve (AuC) of the plot of indifference points against time (i.e., *the higher the AuC, the lower the steepness/rate of discounting*) (Myerson et al., 2001). To reduce floor/ceiling effects, participants who discounted less than 5% or more than 95% after a delay of 5 months in more than one condition were removed.

## Results

After screening participants for the exclusion criteria (8 due to TD criteria, 5 due to non-native speaker criteria), 63 participants (34 females, 29 males; age: *M* = 23.8 years, *SD* = 1.38) remained. Due to the direction of predicted effects, results of planned *post-hoc* comparisons are reported at the 1-tailed level. IRI questionnaire data were lost for two participants due to a technical fault.

To test that individuals exhibited TD, a one-way repeated measures ANOVA was performed with delay as a within-subjects factor and indifference points in the self-condition as a dependent variable. A significant effect of delay was observed, *F*_(5, 310)_ = 180.56, *p* < 0.001, η^2^_p_ = 0.477.

**Figure 3:**
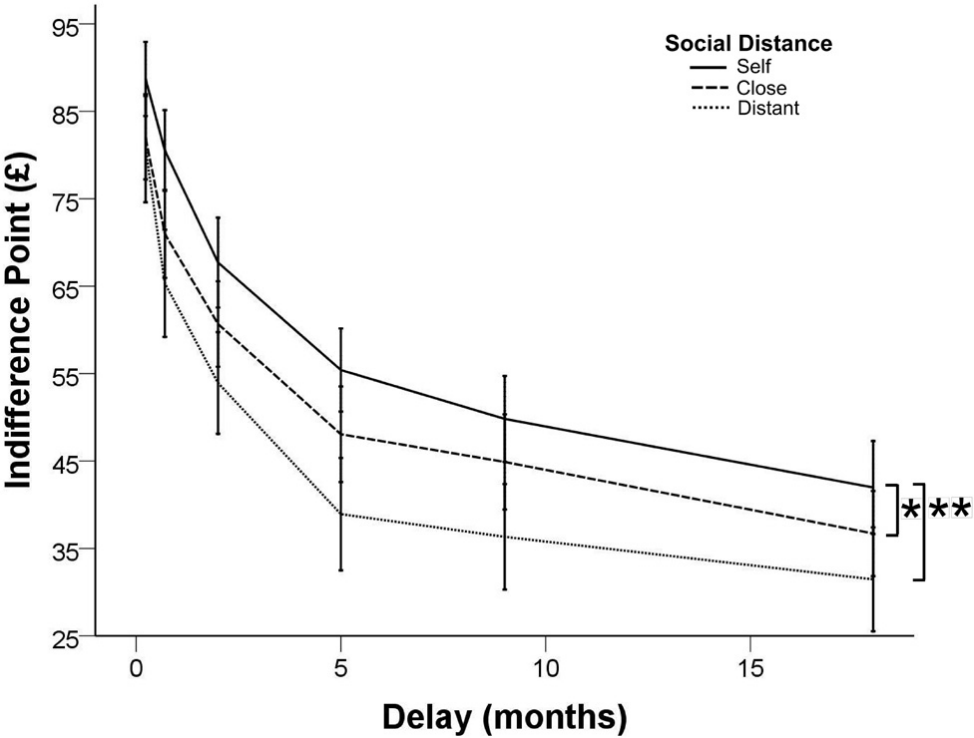
**Temporal discounting curves for social conditions.** Error-bars at 95% CI and adjusted for within-subjects variance (significantly different at: ^*^0.05 level; ^**^0.01 level).

To test the effect of social distance on TD, a one-way repeated measures ANOVA was performed with social condition (self/close/distant other) as a within-subjects factor and rate of TD as a dependent variable. A significant effect of social distance was observed, *F*_(2, 124)_ = 5.12, *p* = 0.007, η^2^_p_ = 0.076. Planned contrasts (Bonferroni corrected) showed a significant difference between self (*M* = 958, *SD* = 58.92) and distant other (*M* = 731, *SD* = 62.39), *t*_(62)_ = 3.07, *p* = 0.003, *d* = 0.39. A marginally significant difference was seen for TD between self and close other (*M* = 850, *SE* = 62.18), *t*_(62)_ = 1.91, *p* = 0.06, *d* = 0.24, but not significant between TD for close and distant others [*t*_(62)_ = 1.48, *p* = 0.143, *d* = 0.24] (Figure 3). Pearson's correlations were performed to examine the association between the steepness of discounting and trait empathy in specific task conditions (see Table 1). Specifically, TD for distant others was negatively associated with empathy scores (i.e., individuals high in empathy discounted less steeply for distant others, *r*_EQ−distant(61)_ = 0.220, *p* = 0.042, *r*_IRI−distant(59)_ = 0.310, *p* = 0.008). No association was found for self [*r*_EQ−self(61)_ = 0.027, *p* = 0.416, *r*_IRI−self(59)_ = 0.050, *p* = 0.352], or close conditions [*r*_EQ−close(61)_ = 0.162, *p* = 0.103, *r*_IRI−close(59)_ = 0.134, *p* = 0.152].

**Table 1:** Correlations between temporal discounting in each social condition and trait empathy measures (significantly correlated at the: ^*^0.05 level; ^**^0.01 level).

		**Self**	**Close**	**Distant**
EQ	R	0.027	0.162	0.220^*^
	Sig.	0.416	0.103	0.042
IRI	R	0.05	0.134	0.310^**^
	Sig.	0.352	0.152	0.009

## Discussion

Making decisions on behalf of others is a common part of everyday life, and yet how we make these choices remains largely unknown. In this experiment, we tested the hypothesis that this process is influenced by social distance and trait empathy. Choice behavior was operationalized using a TD paradigm, where participants were asked to choose between a series of immediate and distant rewards on behalf of close and distant others, as well as themselves. We found that (1) people discount less steeply for themselves compared to others, and that the steepness of discounting for others increases with social distance, (2) compared to people who score low on trait empathy, highly empathic people discount less steeply for distant others.

TD was steeper as the social distance of reward recipients increased across conditions of self, close and distant others. Participants were explicitly instructed to simulate the reward recipients in the task (i.e., “put yourself in the shoes of the recipient”). The ease of simulation varies as a function of social distance, i.e., simulation is easiest when one is making choices about oneself, and participants were found to discount least steeply in this condition. People who are similar to one's self, or who are socially close, are easier to simulate compared to socially distant others. Consistent with this, rewards for self-similar persons are found to be higher in subjective value and show higher activation of reward-related brain areas, when compared to those for self-dissimilar persons (Mobbs et al., 2009). Finally, simulation is most difficult when making choices on behalf of a distant other. As expected, discounting for distant others produced the steepest slope. These parallel findings show that people tend to choose more immediate compared to delayed rewards as psychological connectedness with the reward recipient reduces (Bartels and Rips, 2010).

In this experiment, participants always performed TD for self-last to avoid reported self-bias effects (i.e., the increased tendency to use one's own preferences as a default in choosing for others after having made the same choices for self). It is possible that such biasing effects may work both ways, such that choices for oneself made after choices for others are biased toward others' predicted preferences. However, our results are concordant with those observed by Beisswanger et al. (2003), who used a between-group design to avoid order effects, and reported that choices for others were more impulsive compared to choices for self. Given that impulsive choices are associated with steeper TD (Alessi and Petry, 2003), this supports the present finding that intertemporal choices are more impulsive for others than for self.

Our results contrast with a recent report by Ziegler and Tunney (2012), which shows that choices for others in a TD paradigm become less impulsive as social distance increases. Critically, participants in their task were instructed to select the option that another *should* select. This frames the choice in a way that biases participants toward self-control (i.e., choosing the reward that is best for the recipient, which may not be the reward the recipient would choose on his/her own). This key difference in the frame of operation for the TD task can potentially explain the divergent results.

Simulation is a key empathic mechanism for internally representing the emotions and motivations of others (Keysers and Gazzola, 2007). Accordingly, we expected that the individuals who are high in trait empathy would be able to simulate distant others more easily, and hence discount less steeply when making choices on their behalf. This hypothesis was supported as trait empathy (using two separate trait measures) was inversely related to the steepness of TD for distant others. This finding replicates a previous report showing that people who score higher in trait empathy make more self-similar choices for others (Faro and Rottenstreich, 2006). Additionally, the results suggest a role for empathy in making intertemporal choices, which was elegantly predicted by Loewenstein almost two decades ago (Loewenstein, 1996). This result is also consistent with previous work that suggests a link between TD and other social behaviors. The steepness of TD is negatively correlated with altruistic tendencies (Harris and Madden, 2002; Yi et al., 2005). Steeper TD has also been reported in persons with social anxiety (Rounds et al., 2007), a trait marked by the reduced motivation to affiliate with others (Mallott et al., 2009).

In our study, no significant association was observed between TD for self/close other and trait empathy. This would be expected if the value of delayed rewards for recipients is indexed by individual differences in how easily we can simulate them. This null finding replicates a previous report in which a positive association between trait empathy and less-impulsive choices was noted for others, but not for self (Faro and Rottenstreich, 2006). We speculate that there are two possible reasons why this null finding was observed. First, both trait measures of empathy (IRI and EQ) ask questions about hypothetical unknown/distant others, which increases the sensitivity of these measures over larger social distances. Secondly, the self and close other conditions might be susceptible to ceiling effects in simulation; effects that compress individual differences and make their association with trait empathy difficult to observe. A possible method to overcome this limitation could be to use longer delays in the TD task, reducing the ease of simulation for future selves as done by Bartels and Rips (2010). A caveat of the current study is that the observed relationship between empathy and TD for others may not generalize to the entire lifespan, since the age range of the current study is fairly narrow. Future research should examine this relationship in other age groups, particularly adolescence, when immature discounting is observed (Christakou et al., 2011; Sharp et al., 2011). A second direction for future work is to test the hypothesized role of simulation in intertemporal choices for others using objective indices of simulation measured by psychophysiological and neuroimaging techniques. Current experiments in our lab are testing this.

In this experiment, we show how social distance influences choice of future rewards for self and others, by showing that people discount least steeply for themselves, and most steeply for distant others. We interpret this using a simulation based account of empathy that suggests that socially distant people are most difficult to simulate. Crucially, we find that trait empathy influences how we choose rewards for others; highly empathic people discount less steeply for distant others. Future research should examine these processes in psychopathological populations with deficits in both reward and empathy processes, such as people with addiction (Gizewski et al., 2012), attention disorders (Marton et al., 2008) and those diagnosed with Autism Spectrum Conditions (Schmitz et al., 2008; Dichter et al., 2010; Scott-Van Zeeland et al., 2010).

### Conflict of interest statement

The authors declare that the research was conducted in the absence of any commercial or financial relationships that could be construed as a potential conflict of interest.
